# A Qualitative Scoping Review of the Impacts of Economic Recessions on Mental Health: Implications for Practice and Policy

**DOI:** 10.3390/ijerph19105937

**Published:** 2022-05-13

**Authors:** Olivia Guerra, Vincent I. O. Agyapong, Nnamdi Nkire

**Affiliations:** 1 Department of Psychiatry, Faculty of Medicine and Dentistry, University of Alberta, Edmonton, AB T6G 2B7, Canada; nnamdi.nkire@albertahealthservices.ca; 2 Department of Psychiatry, Faculty of Medicine, Dalhousie University, Halifax, NS B3H 2E2, Canada; vincent.agyapong@nshealth.ca

**Keywords:** economic recession, mental health, qualitative, descriptive, scoping review

## Abstract

In a follow-up to our 2021 scoping review of the quantitative literature on the impacts of economic recessions on mental health, this scoping review summarizes qualitative research to develop a descriptive understanding of the key factors that transmute the socioeconomic stressors of a recession into poorer mental health. The previous study identified 22 qualitative studies from 2008 to 2020, which were updated with search results from six databases for articles published between 2020 and 2021. After inclusion and exclusion criteria were applied to the total 335 identified studies, 13 articles were included. These were peer-reviewed, qualitative studies in developed economies, published from 2008 to 2021, and available online in English. Participants perceived that financial hardship and unemployment during recessions increased stress and led to feelings of shame, loss of structure and identity, and a perceived lack of control, which increased interpersonal conflict, social isolation, maladaptive coping, depression, self-harm, and suicidal behavior. Participants struggled with accessing health and social services and suggested reforms to improve the navigation and efficiency of services and to reduce the perceived harms of austerity measures. Providers should screen for mental distress and familiarize themselves with health and social resources in their community to help patients navigate these complex systems. Policy makers should be aware of the potential protective nature of unemployment safeguards and consider other low-cost measures to bolster mental health supports and informal social networks. Research in this area was limited. Further research would be beneficial given the impacts of the ongoing COVID-19 recession.

## 1. Introduction

Beginning with the World Health Organization’s declaration of the SARS-CoV-2 (COVID-19) pandemic on 11 March 2020, economies worldwide have been rocked by market closures and supply chain, trade, and finance interruptions, leading to global economic recessions [1,2]. The World Bank warned in their 2020 Global Economic Prospects report of a lost decade or more of per-capita income gains and concerns that cumulative factors, including massive public and private debt, would lead to a prolonged deterioration in economic prospects [3]. In June 2021, the World Bank predicted an expansion of 5.6% in the global economy, marking the fastest post-recession pace in 80 years [4]. However, this recovery has been uneven—accounted for primarily by advanced economies—as the slow pace of vaccinations and rapid spread of the Delta and Omicron variants continue to increase uncertainty about how quickly this pandemic can be overcome [4,5]. Rapidly rising inflation rates in many advanced economies reflective of pandemic-related supply–demand mismatches and higher commodity prices are reflected in a downgrade in the economic forecast released by the International Monetary Fund (IMF) in October 2021, which reflected a downgrade in growth to 4.9% in 2022 [5]. In addition, issues of subdued employment growth, housing affordability, food insecurity, rising interest rates, and the challenges of climate change will continue to place financial strain and economic burden on individuals and economies around the world in 2022 [5,6].

In this context, concerns around the implications on population mental health of the ongoing pandemic and related public health restrictions, as well as the lingering economic recession, ongoing global economic uncertainty, and an uneven, faltering economic recovery have become areas of increased public and academic discourse. Prior research in disaster mental health has demonstrated that following both natural and human-made disasters, specific psychological problems have been seen to occur, such as depression, anxiety, and trauma-related disorders [7,8,9]. In a 2021 scoping review of quantitative research on the impacts of economic recessions on depression, anxiety, trauma-related disorders, and illness outcomes, Guerra and Eboreime found that a significant relationship was observed, in which during and following periods of economic recession, increases in depressive symptoms, self-harming behavior, and suicide occurred [10]. Limited or inconclusive data were found to support the relationship of economic recessions with traumatic disorders and symptoms of anxiety [10]. In fact, much of the research in this area since the 2008 global economic recession in advanced economies, and indeed during the COVID-19 pandemic to date, has focused on quantitative analyses of mental illness, primarily among working-age adult populations, leaving considerable room to further develop a broader understanding of how these observed changes in mental health transpire [10].

This follow-up scoping review of the qualitative literature seeks to summarize how people experience the impacts of economic recessions on their mental health in democratic, free-market, first-world nations and to develop a descriptive understanding of the key factors that transmute the financial and socioeconomic stressors into poorer mental health outcomes during an economic recession. The goal is to better inform clinicians and policy makers striving to improve population health surveillance and develop targeted support measures to prevent a mental health crisis in the wake of the ongoing global COVID-19 pandemic and its attendant economic recession.

## 2. Materials and Methods

This is the second part of a two-part scoping review of the mental health impacts of economic recessions. These two reviews mostly cover the same era and topic, but each part is distinct in the papers included, with the first review by Guerra and Eboreime (2021) including quantitative study papers and this second study including qualitative studies. Much of the literature search for this study was completed in the previous review, with 22 qualitative studies from 2008 to 2020 identified by Guerra and Eboreime (2021) incorporated into this current review, which were assessed beside additional articles from 2020 to 2021 identified through the literature search strategy below for inclusion in this current scoping review [10]. Please see Guerra and Eboreime (2021) for the detailed methodology and search history [10].

To update the qualitative body of literature identified during our previous scoping review, this follow-up study utilized a comprehensive search of databases PsycINFO, MEDLINE, Embase, Web of Science: Core Collection, National Library of Medicine PubMed, and Google Scholar completed between 24 and 26 November 2021 to identify relevant articles. This is in keeping with Bramer et al.’s 2017 study on optimal search strategies for conducting systematic reviews [11]. The following search terms were used: “economic recession” AND (“depressive disorder” OR “depression” OR “major depressive disorder” OR “major depression” OR “MDD” OR “generalized anxiety disorder” OR “generalized anxiety” OR “GAD” OR “anxiety” OR “post-traumatic stress disorder” OR “post traumatic stress disorder” OR “PTSD” OR “mental health” OR “mental illness”). Articles were limited to publication dates from the date that each database was searched in the 2021 scoping review conducted by Guerra and Eboreime to the present, as allowed by the database search filters [10]. For the databases PsycINFO, MEDLINE, Google Scholar, and Embase, articles were limited from 1 January 2020 to the present (24–26 November 2021). Results from the National Library of Medicine Pub Med were limited from 29 November 2020 to the present (24 November 2021) and for the Web of Science: Core Collection the search articles were limited from 20 December 2020 to present (24 November 2021). In addition, all results were limited to English only and full text articles. All citations were imported into EndNote, which was used to sort and store article references.

Duplicate records were removed prior to screening. Inclusion criteria for articles were qualitative studies of all ages, conducted in Organization for Economic Coordination and Development (OECD) countries, assessing mental disorders during periods of economic recession. Exclusion criteria included articles not relevant to the research question; results that were opinion, editorial, commentary, or non-peer reviewed pieces; literature reviews; and those that used quantitative methodologies or were conducted in non-OECD countries, as these did not address the research question. Each article abstract was reviewed by the primary author (OG) for suitability based on the above inclusion and exclusion criteria. During secondary screening, the full texts of all remaining studies were read by the primary author (OG) and their final eligibility was assessed.

The included articles were then summarized in a Microsoft Excel spreadsheet and sorted alphabetically by primary author, collating the study information and results for each article, including the recession period studied, study population, methodology employed, and key findings related to the research question. These data points were selected to most efficiently summarize the diverse studies included using these search parameters. Finally, key findings of each article were coded by the author into overarching themes to create a narrative summary of the findings that emerged from the body of literature included in this review.

No assumptions or simplifications for data variables of the collated search results were made. A critical appraisal of the literature and sources of evidence was not completed during this review, as the aim of a scoping review is to explore the breadth of information available, rather than to assess the relative strength of findings or recommendations.

## 3. Results

**Figure 1:**
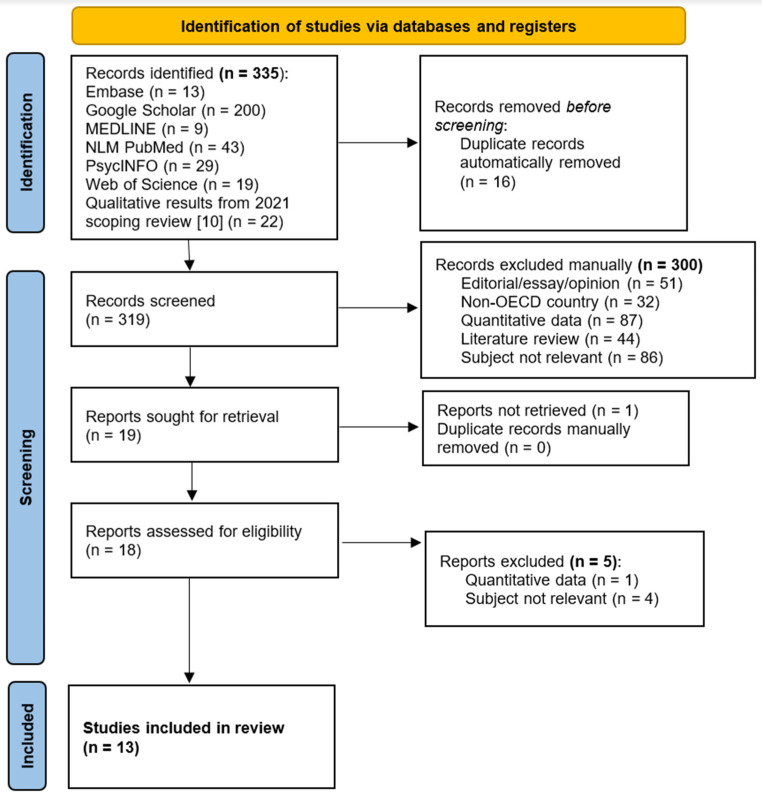
PRISMA literature search flowsheet [12].

Using the above search parameters, a total of 335 articles were initially included and imported into EndNote citation manager. After 16 duplicate records were removed, a total of 319 articles were reviewed by abstract and a further 300 were excluded. See Figure 1 for full details of the literature search and review [12]. One report was not retrievable, resulting in a final body of 18 articles being assessed for eligibility in secondary screening, of which 13 articles were included in this systematic review. A flowchart of the literature search can be seen in Figure 1.

Of the 13 articles included, the majority were completed in Europe, with five studies from the United Kingdom (UK; 38.5%), three from Portugal (23.1%), two from Spain (15.4%), and one from Sweden (7.7%). One study was completed in each of Canada (7.7%) and the United States of America (USA; 7.7%). All included studies were qualitative in nature, although three articles employed a mixed qualitative and quantitative methodology (23.1%). Six utilized semi-structured in-depth interviews (46.2%), three used a combination of semi-structured interviews and focus groups (23.1%), two combined a quantitative survey with focus groups (15.4%), one used focus groups alone (7.7%), and one employed a mixed quantitative and qualitative questionnaire (7.7%). A summary of the study population, methodology, and key findings for the 13 included articles can be found in Table 1.

The topics covered in the included studies were heterogeneous, and no standardized, validated interview or focus group guide was used in any study. Each relied on question or topic guides developed by the researchers, and in three cases with input from target population advisors. Nonetheless, the breadth of topics covered overlapped between many of the studies, as seen in Table 2.

Study populations in all articles were adults over the age of 18 years and focused on targeted populations. These included healthcare providers (HCPs) (30.1%), people who self-harmed or experienced suicidal ideation related to socioeconomic changes (23.1%), unemployed parents or adults (23.1%), primary healthcare users (15.4%), people experiencing socioeconomic changes who did not self-harm (7.7%), adults over age 65 years (7.7%), people with severe and persistent mental illness participating in individual placement support (IPS) services (7.7%), unemployed autoworkers (7.7%), and immigrant workers (7.7%). (Please note, several studies included more than one of the above study populations; therefore, the total of the percentages provided does not add up to 100%.).

Study populations were recruited using convenience sampling in four studies (30.8%); purposive sampling in four studies (30.8%); snowball sampling in one study (7.7%); or any combination of convenience, purposive, and snowball sampling in four studies (30.8%). Study population sizes were relatively small, in keeping with qualitative methodological research, and ranged from 16 to 73 participants.

## 4. Discussion

### 4.1. Changing Socioeconomic Conditions and Mental Health

During periods of economic recession, unfavorable changes in the labor market resulting in layoffs, precarious work conditions, decreases in salary or wages, and pension or benefit cutbacks were correlated by participants with increased levels of stress and negative emotions [14,15,16,17,19,20,21,22,23,24,25]. For those who remained employed, working conditions were noted to deteriorate with decreased working hours, increased workload, decreased occupational health and safety standards, increased part-time or contract work, and increased competition for existing jobs, leaving many people feeling that they had to accept whatever conditions of employment were offered to them [15,16,18,25]. Negative mental health outcomes described by participants included feelings of stress, worry, fear, anxiety, irritability, hurt, frustration, anger, boredom, worthlessness, shame, sadness, and depression [14,15,16,17,19,20,21,22,23,24,25]. There was a correlation reported by three studies with self-harm and suicidal behaviors among people affected by economic crises in the UK and the USA [15,16,20]. These changes were seen across the included study populations [13,14,15,16,17,18,19,20,21,22,23,24,25].

Downstream consequences of these shifts in mental well-being that were noted by HCPs in Portugal and Spain included increased absenteeism, sick leave usage, and early retirement, which led to higher levels of income loss amongst these patients [14,19]. Although HCPs questioned whether there was an overall increase in the incidence of major psychiatric disorders, they observed negative repercussions in addition to decreased mental well-being, such as worsening of existing physical ailments, problems with sleep, increased presentations of psychosomatic problems, malnutrition, and increased suicidal behavior and completed suicides [14,19,25]. These consequences were observed by study participants to accumulate among those most vulnerable either financially or socially prior to the recession, namely those with lower socioeconomic status, adults on a fixed income (older adults and those on disability benefits) or with high levels of debt, immigrant workers, blue collar workers, and people with a history of mental health conditions or pre-existing mental health vulnerabilities (such as a history of childhood trauma or adverse childhood events (ACEs)) [14,16,20,21,25].

Participants perceived economic changes as being translated into negative mental health outcomes through financial strain or hardship, which resulted in decreased access to essential goods (e.g., food, medicine, health services, etc.), decreased capacity to make bill payments, and housing instability [14,15,16,20,21,23,25]. Studies also found that participants reported their worries about the future as equally powerful drivers of stress and anxiety beyond the actual economic consequences experienced [15,16,20,21,22,23,24,25]. These worries included their ability to find work, consequences of increasing debts, threat of eviction or foreclosure, fear of further benefit cuts or sanctions, consequences for themselves and their families, and possible consequences of unstable work on maintaining work permits or residency status for immigrant workers [15,16,20,21,22,23,24,25]. For older adults, an additional consideration of declining health resulting from limited economic resources included increasing costs for services that they could previously perform independently, and increased transportation costs and social isolation secondary to decreased mobility [21].

Changes in consumption patterns because of financial strain were cited as a significant stressor leading for some to major consequences (e.g., malnutrition, decreased access to medications and healthcare, or housing instability), while for others reduced budgets required only limiting leisure activities, such as shopping, attending social events, or travelling [14,15,16,17,20,21,22,23,24,25]. Although the latter may seem comparatively insignificant, increased levels of shame secondary to perceived failures in social roles, as well as increased social isolation due to lack of participation in leisure activities, were seen as a major contributor to poor mental health across studies [15,16,20,21,22,23,24,25].

Three studies referred to the idea of “unemployment stigma”—or the belief that it is necessary to have a job to fulfill the role of “a good person” in society—and that although the stigma was primarily internalized or only implied by others, it remained a powerful driver of behavior among study participants [15,23,24]. Cutbacks in social activities were experienced as shameful and leading to feelings of inadequacy and becoming a source of significant despair and worthlessness linked in three studies to self-harm and suicidal behavior [15,16,20]. These associations were noted to be worsened by a sense of “extreme self-reliance” observed among the population of study participants who had self-harmed as compared to those experiencing financial hardship in the same community who had not self-harmed [16]. Specifically, those who had self-harmed or were hospitalized for suicidal behavior were more likely to feel that only they could have helped themselves, they blamed themselves for their life circumstances, expressed a reluctance to burden others, described cultural expectations of masculinity that limited their willingness to seek help, and reported more strained relationships with family and informal supports [16,20].

Loss of employment was experienced as a loss of identity, structure, motivation, or “purpose in life”, and for some participants work was felt to be the basis for belonging and dignity as a human being that gives structure and security to life [23,24]. Consequently, the inability to find work led many to experience frustration, impotence, and a lack of control that led to a sense of despair [15,23]. Even for those for whom unemployment was initially viewed as positive or who viewed the future with optimism, as the duration of unemployment increased, feelings of hopelessness and uncertainty for their future also increased [24]. Receiving no response from employers was experienced as particularly harsh, leading to a lack of hope for the future, diminished self-esteem, and use of maladaptive coping strategies, such as drugs, alcohol, and self-harm [15].

Ten studies reported on changes in romantic or familial dynamics because of periods of economic recession. Deteriorating romantic or family relationships were linked by participants to negative mental health outcomes in eight studies [14,15,16,20,22,23,24,25]. In three studies, parents expressed concerns about the impact of their own unemployment, financial hardship, and consequent increased tension in the home on their children’s emotional well-being [2,3,4,5,6,7,8,9,10,11,12,14,15,16,17,19,20,21,22,23,24,25]. Some parents perceived a threat to their children’s education and future observed through behavioral changes (sadness, worry, irritability), increased problems at school, and diminished access to leisure or educational activities [17,22,25]. For parents of young adults who were laid off during the financial crisis in Portugal, the return of adult children to the parents’ home resulted in an increase in parents’ financial hardship and subsequent loss of well-being [14]. HCPs in Spain observed that some patients were suffering health problems owing to an obligation to assume greater responsibility in caring for dependents following cuts to social care budgets and insufficient household resources to pay caregivers or care homes [19]. Among immigrant workers, gender disparities in loss of employment left many marriages in distress after husbands were more frequently laid off compared to their wives [25]. In two studies, unemployment conditions were associated with marital crisis, increased sexual problems, family tensions, and even relationship breakdowns [17,25]. In addition, immigrant workers reported seeing the negative effects on their children’s quality of life as a “failure of a life project” and expressed worry about economic dependents in their country of origin [25].

### 4.2. Access to Resources during Recessions

In five studies, participants commented on the availability of mental health supports during the economic recession [16,17,19,20,24]. In total, 55.9% of laid-off Canadian autoworkers reported limited access to community mental health resources, and patients admitted to a public pay hospital in the USA following suicidal behavior reported that job loss resulted in decreased access to mental health care (37.5%), prescription medication coverage (37.5%), and medication management (31.3%) [17,20]. Notably, of the 13 articles included in this study, 12 were completed in countries with universal healthcare provision, except for the USA; therefore, it is difficult to determine eventual differences between services and supports conducted in countries without universal healthcare. However, despite living in countries with publicly funded and universally accessible healthcare services, Canadian autoworkers and unemployed adults in Sweden reported endorsed a reluctance to access services due to financial constraints [17,24].

In Barnes et al.’s 2017 study, they found that many people who self-harmed only accessed support from their general practitioner (GP) after harming. When they did attend, a positive GP experience involved having a consistent, supportive relationship with the physician, feeling they had time to talk and were properly listened to, and that they received useful information about psychological support services [16]. Most participants wanted at least an offer of talk therapy and many sought medications for depression, stress, anxiety, and insomnia secondary to concerns over debt, benefits, or financial difficulties [16]. Challenges with navigating mental health resources cited by participants included an overall lack of resources, long wait times, and waiting list errors [16].

In several studies, participants also reported difficulties with access to practical, clear help for their financial difficulties [15,16,18,21]. In one study, the most common services accessed by study participants were not health services, but rather employment-related services, such as job centers and benefits agencies, and financial advice services. However, when trying to access social services, many people found a paucity of updated information on community resources, poor referral routes between services, long wait lists, and difficulties with service provider funding due to short-term grants that limited the continuity of supports once users were connected [16]. Among specific populations, such as those with severe and persistent mental illness (SPMI) and older adults, researchers noted difficulties with accessing financial support and public benefits through the convoluted public system and noted that older adult patients may in fact overestimate their financial capabilities [18,21]. They advised that HCPs should be wary when services such as “meals on wheels” or luncheon clubs are cancelled, as such changes may be due to a lack of finances rather than a lack of need [21].

For some, the behavior of job center staff was noted to be poor and felt to be stigmatizing, which one job center employee believed was a result of a pressurized system with high quotas and work capacity adding tension to staff members [16]. Similarly, HCPs in Spain reported pressure on both individual and system levels secondary to austerity measures combined with increased demands during the economic recession [19]. They reported cuts in the number and working hours of staff, forced retirements, stricter control of sick leave, increased workloads, and inappropriate use of resident physicians, combined with decreases in equipment, referrals allowed, and funding for research [19]. These tensions were felt by HCPs to decrease quality of care; increase wait times; lead to hospital, primary care, and emergency service overcrowding; and decrease ambulance services, the availability of emergency room services in rural settings, and access to patient transfers for specialist care [19].

Attempts by policy makers to improve system inefficiencies were noted by HCP participants in three studies to increase the vulnerability of patients with low socioeconomic status and those with mental health problems [13,16,19]. In particular, the implementation of user co-payments in Portugal and Spain may have constituted an important financial barrier to individuals with low economic resources but not exempted from the user fees [13,19]. While some HCPs saw these changes as necessary to reduce overuse or inappropriate use of services and decrease no-shows for appointments, others felt that the barriers posed by these fees for some patients had led to serious adverse outcomes [13,19].

Measures such as privatization were largely unpopular amongst HCPs in Spain, who generally regarded private care centers as prioritizing profits and decreasing costs at the expense of adequate patient care, although some division over the efficiencies of the public versus private systems existed between HCPs [19]. Those with a positive view of the private system felt it had better management, less wastage, and provided a similar quality of care to public centers, while those opposed noted issues with early discharge of patients, biased risk selection, and “cream-skimming” of patients to increase profits [19]. Governance issues including corruption and mismanagement were highlighted as concerns at all levels of the health care system (both public and private) by HCPs, with a particular focus on an observed deficiency of planning and ineffective decision making that lacked engagement with service providers, associations, and citizens in policy decisions [19].

### 4.3. Individual Resilience Factors and Systems-Based Solutions

Studies not only addressed vulnerabilities that increased the risk of accumulation of adverse events during periods of recession, but six studies also highlighted individual resilience factors linked to better outcomes during periods of financial hardship [16,20,21,22,23,24]. In general, people’s social capital—or informal supports who could provide both practical and emotional support—had significant mitigating effects against poor mental health and outcomes such as self-harm and suicidal behavior [16,20,21,22,23,24]. Specific offerings such as assistance with meals, paying bills, pooling resources, socializing, helping one another, and providing emotional support were observed to be helpful [16,21,22,23]. Additionally, the amount and nature of support received were observed to limit the extent of reduced agency experienced by those who were involuntarily unemployed [23]. Amongst American patients hospitalized for suicidal behavior, the greatest benefits of admission perceived by patients included having loved ones and hospital staff show concern, realize the patient’s levels of distress, and increase their motivation to change relationship dynamics emphasizing the deep importance of social capital and relationships [20]. Having a valued ‘fall back’ social role, like parenting or volunteering, was found to be protective against the identity disruption of job loss, and while financial hardship can increase the strain on marital and parent–child relationships, family members can also be brought closer together by these conditions as a mechanism of social support [22,23]. These studies underscore the value of healthy family ties as a form of social capital.

Other individual resources observed included activities, structure, and affiliation, which provided people with meaning, hope, and purpose [24]. Among older adults, an optimistic perspective and lifelong approach to budgeting, an aversion to credit, and an ability to make ends meet and to financially plan were all linked with resilience, as these factors offered individuals some control over their expenses during times of hardship [21]. Finally, people with more knowledge of free debt advice organizations and how to access them were seen more among the healthy community sample compared to those that had self-harmed in the UK, suggesting the value of access to financial supports during difficult times [16].

At a systems level, suggestions for improvements revolved around increasing the ease of access to financial and mental health services. Suggestions included improving channels of communication between users and administrative staff, providing clear, practical financial supports, and increasing awareness of and access to the same through centralized contact points that would coordinate and refer people to appropriate services and decrease wait times [13,15,16]. Studies suggested cross-training between financial support services (free debt assistance centers, job centers, etc.) and mental health services, or an integration of both service systems, which would help staff recognize and refer users to the appropriate supports when required and improve communication between providers [13,17,22]. In addition, they suggested that community partnerships and interventions sensitive to local population needs could assist with prevention and health promotion initiatives, increase the use of available technology to improve communications, and develop infrastructure to improve service access [13].

Both patients and HCPs advocated for increased investment in primary care and mental health services to increase human resources, decrease wait times, and provide for timely referrals from primary care to psychiatric services [13,17]. They advocated for educational initiatives regarding the appropriate use of healthcare services to replace measures meant to increase system efficiencies that were felt instead to create barriers to patients, such as co-payments or user fees [13,19]. Within existing healthcare systems, suggestions for improving efficiencies included targeted hiring of young HCPs on permanent contracts, improving working conditions to decrease burnout and attrition rates, use of evidence-based guidelines, optimizing resource use, decreasing wastage, increasing productivity with performance measurements and auditing, clarifying leadership structures and accountability of health and administrative professionals, increasing participation in decision-making via consultation with HCPs, stopping privatization of health care, and public health tracking and surveillance of disease and states of health [13,17,19].

Regarding access to social services, study participants advocated for government-driven approaches to increase the availability of jobs and income redistribution programs, as well as reversal of benefits and pension cuts to increase access to health services and therapeutic compliance [13]. Service users wished for employers and job centers to be aware of the harm conferred by unresponsiveness to job applications [15]. The most helpful supports received through free debt advice centers were specific services such as staff addressing or translating dense and confusing communications from benefits agencies, banks, and creditors, acting as an advocate for the user, or giving information about how to prioritize and manage debt [16]. Practical barriers, such as literacy problems or limited language skills, with the process of job applications and form filling for accessing benefits should also be considered when evaluating and prioritizing access to supports [16].

### 4.4. Practice and Policy Implications and Future Directions

During periods of economic recession, the populations most affected by mental health concerns, including depression, self-harm, and suicide, include men approaching retirement age and people with low education, high levels of unemployment or job insecurity, and low pre-recession socioeconomic status [10]. Unfortunately, prior studies have shown that these are not the populations most likely to access mental health supports [26,27]. Additionally, people who commit suicide related to economic recession are less likely to access the family physician or mental health supports or be formally diagnosed with mental illness prior to suicide compared to non-recession-related suicides [28,29,30,31,32]. This is in keeping with the suggestions of qualitative studies in this review, namely that those who self-harmed or attempted suicide related to economic recessions were more likely to be extremely self-reliant and poorer in terms of social capital, and thereby less willing and able to seek out both formal and informal supports [15,16,20].

Clinicians should routinely explore mental health concerns and facilitate access to mental health and social services as needed for patients with high levels of vulnerability, including pre-existing mental health concerns, a history of adverse life events, high levels of self-reliance, limited social supports or poor social capital, recent or prolonged unemployment, financial hardship, and social isolation [14,15,16,17,20,21,22,23,24]. HCPs should be aware that presenting complaints may appear initially unrelated to these changing socioeconomic conditions, however concerns around psychosomatic issues, worsening of pre-existing health conditions, chronic pain, insomnia, non-specific stress, worry, irritability, anxiety, and interpersonal conflicts were also correlated with the stress from economic recessions and poorer mental well-being [14,15,16,17,19,20,21,22,23,24,25].

Barriers to access both health and social services should be minimized and further investment in these realms may be warranted to help services accommodate the influx of users that can be expected during nationwide or global recessions [13,16,17,20,21,22]. Several participants across studies did not recognize their need for mental health or general health supports and were more focused on access to financial or social services, suggesting the importance of cross-agency awareness of these concerns and timely referral between health and social services to prevent negative health outcomes [15,16,17,19,20,21,22,24,25]. It may also be prudent to focus public educational efforts to increase vigilance to identify people in need of support at the community level, among places of employment, unemployment, or income support offices.

During the ongoing COVID-19 pandemic, policy makers should consider reinvestment in unemployment protections and labor programs as mitigating factors in reducing the negative social and mental health impacts of economic recessions, including depression, self-harm, and suicide mortality [10,33,34,35]. However, like recommendations made in our previous study, due to the inherent challenges in increasing resources during the budgetary restrictions of a recession economy, governments should also consider the adoption of low-cost, evidence-based interventions such as bibliotherapy, Internet-delivered cognitive behavioral therapy, supportive text messaging, and encouragement of community- and family-level emotional support [10,36,37,38,39,40,41,42,43,44,45,46,47,48,49,50,51,52,53,54,55,56,57,58,59,60]. As described in this review, individuals with high social capital and strong informal support networks reported less severe mental health impacts of the financial hardships endured during times of economic recession, suggesting that bolstering the family unit and social cohesion through public health promotions, supportive psychotherapeutic options, and other such low-cost interventions could be beneficial at the population level during periods of economic recession [16,20,21,22,23,24].

Future areas of research could consider studying such low-cost interventions, as well as policies to increase primary care access, coordination of services, and public awareness. Overall, this remains an understudied area of mental health research with far-reaching consequences in our modern world; therefore, further qualitative explorations of the contexts, lived experiences, and connections between periods of recession and increased levels of stress, anxiety, depression, self-harm, and suicide remain urgent in future studies. Future research could also explore how different economic recessions differentially impact societies and individuals and may lead to differential outcomes in mental illness, self-harm, and suicide. For example, the 1987 stock market crash, 2008 housing foreclosure and ensuing global recession, and the COVID-19 pandemic recessions have distinct etiologies and associated stressors that may differentially impact mental health at both individual and population levels, and may benefit from different response strategies.

### 4.5. Strengths and Limitations

The results of this review are limited by the small body of research meeting inclusion criteria for this article. In addition, the possibility of evidence selection bias present in any review study was increased here by having a single reviewer of the literature to apply inclusion and exclusion criteria. Within the body of literature included there was a large degree of heterogeneity between sample populations and a lack of standardization of interview and focus group questions. The articles included are largely Eurocentric, thereby limiting the generalizability of this information, even amongst OECD nations.

Causal differences between the economic recessions of 2008–2009 and the 2012–2015 periods across Europe and the recession caused by the current COVID-19 global pandemic limit the application of findings from the research presented in this review to current concerns. For example, the 2008 global economic recession was largely driven by the collapse of the housing market in the USA, while the current pandemic-driven recession is complicated by mental health impacts not only due to unemployment and financial strain, but also social isolation, health anxiety, and restrictions of freedoms secondary to the spread of the SARS-CoV2 virus and subsequent public health restrictions [5,6].

We note that the small body of literature included in this review limits our ability to draw conclusions about the nature of each recessionary period and the direct impacts on the findings of each of the studies included. Instead, we endeavored to present the information for all 13 studies as one body and highlight here that there may be more diversity in real-world experiences of mental health and service implications during times of recession than this body of literature can adequately represent given the relative paucity of qualitative data in this field at present. Although the body of literature included in this review was relatively small, the findings supported the conclusions of our previous quantitative review of the impacts of economic recessions on mental health, suggesting that the practical suggestions made by study participants and researchers in this scoping review may remain relevant considerations during the current recession in the North American context [10].

## 5. Conclusions

This scoping review summarizes the current qualitative literature from OECD nations on how people experience the impacts of economic recessions on their mental health to develop a descriptive understanding of the key factors that transmute financial and socioeconomic stressors into poorer mental health outcomes during these challenging times. In this review, mechanisms perceived by study participants included direct increases in stress levels and worry or anxiety over the practical consequences of decreased income levels due to poorer working conditions, layoffs, benefit or pensions cuts, and unemployment [14,15,16,17,19,20,21,22,23,24,25]. Additional factors included losses of structure, motivation, and identity tied to the loss of employment or increased reliance on public welfare benefits, which were internalized as unemployment stigma and welfare stigma [15,16,20,23,24,25]. Perceived lack of control, shame, and boredom contributed to patterns of increased social withdrawal, interpersonal conflict, isolation, and depression [14,15,16,17,20,23,24,25]. The practical suggestions made by study participants and researchers in this scoping review include suggestions for increased access to health and social services, as well as system-wide policies to increase detection of people in need of supports, remove barriers to care, and increase system efficiencies during times of strained financial resources at all levels of society [13,15,16,17,19,21,22].

Several implications for clinical intervention and policy have been identified in this scoping review to improve public and service provider awareness of these mental health concerns and those at highest risk, to improve service access, and to invest in practical, evidence-based and cost-effective options to protect public mental health during recessionary periods. Given the broad reaching impacts of this subject, particularly during the uneven global economic recovery and ongoing threat posed by the COVID-19 pandemic, further research into the efficacy of such interventions and novel approaches to preventing, identifying, and supporting those at highest risk of mental distress and illness during economic recessions remains a priority.

## Figures and Tables

**Table 1:** Summary of included articles (N = 13).

Authors (Year)	Recession Period	Study Population	Methodology	Key Findings
Antunes, Frasquilho, Zózimo, et al. (2019) [13]	The 2008 financial crisis; the Great Recession (Portugal)	Healthcare professionals (HCP) (N = 27) and patients at primary health centers (N = 26) in Lisbon, Portugal.	Twenty-seven 27 semi-structured interviews with HCPs and five patient focus groups	Focused on solutions to address consequences of the economic recession and subsequent austerity measures. Patients suggested improvement in access, management, and efficiency of health services; social, economic, and living conditions through job availability and better salaries; investment in and recruitment of human resources for healthcare provision; investment in and promotion of mental health care. HCPs suggested improvement in integration, articulation, and coordination of health services; access to and delivery and quality of primary care services; investment in mental health professionals and improved working conditions within the health sector and social, economic, and living conditions through reversal of austerity measures.
Antunes, Frasquilho, Zózimo, et al. (2020) [14]	The 2008 financial crisis and ensuing Great Recession (Portugal)	HCPs (N = 27) and patients at primary health centers (N = 26) in Lisbon, Portugal.	Twenty-seven semi-structured interviews with HCPs and five patient focus groups	Participants saw changes in socioeconomic conditions (unemployment, precarious work conditions, and loss of income) as risk factors for poor mental health (depression, anxiety, sadness, anger, and irritability) mediated by financial hardship and deterioration of family relationships and environment. The return of young adults to their parents’ home because of worsening socioeconomic conditions contributed to parents’ financial hardship. Consequences of experiencing mental health problems during the recession (e.g., disability, sick leave, and early retirement) were perceived to occur more frequently among those who were more socially disadvantaged.
Barnes, Gunnell, Davies, et al. (2016) [15]	The 2007 to 2012 economic recession (United Kingdom (UK))	Patients who had self-harmed and attended hospital in two UK cities between December 2012 and March 2014 (N = 19).	Nineteen in-depth, semi-structured interviews	For many the self-harm episode was precipitated by issues related to the recession, such as losing or being unable to find work, fears or experiences of benefits changes or sanctions, increasing debt and housing difficulties, such as threat of eviction. All participants described other contributors to their despair, which became more salient during the hardships; for example, childhood abuse or neglect, bullying, sexual identity issues, abusive adult relationships, significant bereavements, and longstanding mental health problems. Participants expressed the desire for practical help with their economic difficulties and therapeutic support for co-existing or historical problems.
Barnes, Donovan, Wilson, et al. (2017) [16]	The 2007 to 2012 economic recession (UK)	Three study populations in two UK cities from Sept 2012 to Jan 2015: front-line staff at health and social service organizations (N = 27), individuals who self-reported problems secondary to the recession and who either had (N = 19) or had not self-harmed (N = 22).	Fifteen interviews and two focus groups with front-life staff; 17 interviews and 1 focus group with the community sample, and 19 interviews with individuals who had self-harmed.	Most participants found accessing health and social services could be difficult. Free debt advice was considered the most useful service. The community sample reported more knowledge of how to access debt advice than the patient sample, although both groups had sought similar types of help. Participants who had self-harmed reported fewer sources of support and less supportive social networks than the community sample. They also reported more difficult economic circumstances, such as benefit changes or sanctions. All groups indicated that practical help for financial and benefit issues would have helped/would help, particularly the patient group. All groups wanted straightforward information about available social and mental health services and how to access them, as well as coordination between services. Participants who had self-harmed had a stronger belief that they should be self-reliant in the face of economic and mental health difficulties than the community sample.
Bartfay, Bartfay, and Wu (2013) [17]	The 2008–2009 global economic recession (Canada)	Laid-off blue-collar autoworkers in Durham Region, Ontario, Canada between September and November 2009 (N = 34).	Participants completed a quantitative questionnaire (demographics and financials), followed by semi-structured focus groups with 4–6 participants per group.	All participants reported a high degree of stress, anxiety, or depression. More than half (55.9%) expressed concerns about the lack of mental health resources in their community; 29.4% of participants reported altered sexual function and intimacy due to emotional stress, as well as problems sleeping; 70.6% reported difficulty with effectively managing occupation-related chronic pain and discomfort. Most respondents had ceased going to complimentary healthcare providers for pain management due to financial costs (61.8%) and 32.4% reported difficulties with affording prescription medications. Notable hardships were reported for participants and their family members (38.2%), e.g., children experiencing stress, anxiety, or school problems, or loss of housing due to inability to pay rent/mortgage (21.9%).
Boycott, Akhtar, and Schneider (2015) [18]	The 2008–2009 economic recession (UK)	People with severe mental illness receiving individual placement and support (IPS) services for at least 6 months in the UK (N = 31).	Semi-structured interviews completed between July 2011 and December 2012.	Personal barriers to returning to work identified by participants included mental health symptoms, effects of medication, and disclosure of their mental health history to employers. Overall, the concerns expressed by participants did not differ substantially from those reported in papers where the wider economic situation was less bleak. The most notable differences identified were challenges with competition for jobs or lack of jobs available given the difficult economic times.
Cervero-Liceras, McKee, and Legido-Quigley (2015) [19]	The 2008–2009 European financial crisis (Spain)	Nurses (N = 5) and doctors (N = 16) across specialties in both urban and rural areas in Spain.	Semi-structured face to face interviews between May–June 2013.	The impact of financial crisis and austerity measures were observed by most HCP participants to generate an increase in levels of anxiety and depression with negative repercussions for existing physical ailments and increasing psychosomatic problems. An increase in suicides was reported. Some patients were suffering health problems due to increased caregiving burden. Healthcare workforces were affected by cuts in the number and working hours of staff, forced retirements, wage reductions, stricter control of sick leave, reduced funds for research, inappropriate use of resident trainees, and increases in wait times and overcrowding of hospitals.
Elliott, Naphan, and Kohlenberg (2015) [20]	The 2007–2008 financial crisis in Nevada, United States of America (USA)	Adults 18 or older hospitalized for actual or threatened self-harm, with or without the intent to die at a state-funded hospital (N = 16).	In-depth, semi-structured interviews conducted in 2007.	Participants reported deep disappointment with others (n = 12), extreme financial strain (n = 8), and lack of mental health care or medications (n = 8) as precipitants of their suicidal behavior. All reported economic hardship that they blamed upon themselves; 94% were unemployed at the time of hospitalization, 69% had unstable living situations, 50% were living with and dependent on others, 25% were evicted from their apartments or had their homes foreclosed. All expressed feeling inadequate about their life circumstances and blamed themselves for their situations. For 9 of 12 participants who attempted suicide, interpersonal problems that precipitated their attempts seemed to improve with hospitalization, although none of the participants said they engaged in suicidal behavior with the intent of receiving care.
Fenge, Hean, Worswick, et al. (2012) [21]	The 2007–2008 global economic recession (UK)	Older adults (65 or older) who are ‘asset-rich–income-poor’ living in the UK (N = 28).	In-depth, semi-structured interviews conducted between September 2010 and January 2011.	Extra financial demands from the recession, due to rising prices and reduced incomes, resulting in worry and stress. Linked to the fear of debt and a reluctance to borrow money, as well as worries about coping in the future. Anxiety about finances means that older people re-consider what lifestyle they can afford, reducing social activities because of the expense, ultimately impacting social well-being, increasing loneliness and isolation. In addition, declining health leads to increased costs for services and reduced mobility, which can lead to increased transport costs and social isolation.
Frasquilho, Gaspar de Matos, Santos, et al. (2016) [22]	The 2013 economic recession (Portugal)	Unemployed adults receiving state unemployment benefits with children 10 to 19 years old in Lisbon, Portugal (N = 59).	Questionnaire: demographic data, employment history, and an open-ended question on changes in family life because of unemployment.	Unemployment was seen as a source of economic hardship and pressure, requiring the household to cut down on essential needs (house, food, health), children’s education costs, and family leisure activities. Family relations were negatively affected, resulting in more friction, conflicts, and harsher parenting. Positive changes including more proximity and support were also reported. All participants’ felt unemployment was a source of psychological distress and low well-being, with increased worry, anger, bad temper, and sadness. Youth well-being was less stated, but some participants did perceive increased levels of sadness, worry, and bad temper in their children following parents’ unemployment.
Giuntoli, Hughes, Karban, and South (2015) [23]	The 2008–2010 economic recession (North England)	Adults in Bradford, England who lost their jobs between July 2008 and October 2010, presumably secondary to the economic downturn at that time (N = 73).	Basic health questionnaire and 16 focus groups were conducted with participants between July to October 2010.	Involuntary unemployment was experienced as a divestment passage for all study participants with themes emerging of: reduced agency related to financial strain and difficulties in finding a new job; disruption of role-based identities related to personal identity crises and loss of time structure and motivation; and experiences of ‘spoiled identities’ related to unemployment stigma and welfare stigma. Reduced agency was felt as a diminished capacity to pursue one’s goals and plans. Disruption of role-based identities had an immediate effect on the participants’ hedonic and psychological well-being, particularly in the form of negative evaluations of self-realization. Unemployment stigma was primarily felt in the context of family relationships, primarily through self-stigma rather than experiences of direct discrimination.
Hiswåls, Marttila, Mälstam, et al. (2017) [24]	The 2008 economic recession (Sweden)	Adults (18+ years) in Gävle, Sweden registered at an employment agency having become involuntarily unemployed for at least six months during or after 2008 (N = 16).	In-depth, semi-structured interviews.	Respondents described work as an important part of identity and the basis for belonging. A poorer financial situation was described as touch to deal with, creating worry, stress, and insecurity. This contributed to feelings of alienation and inadequacy impacting social life and consumption patterns. Therefore, this led to social isolation, which then reinforced emotions of worthlessness and shame and reduced the chances of establishing new contacts and connections that could lead to a job. Problems with sleep and progressive pain were triggered by hopelessness, fear, and anxiety. Respondents who initially saw unemployment as a positive or remained optimistic became more hopeless and uncertain as the duration of unemployment continued. Activities, structure, and affiliation in other contexts (e.g., exercise, activities that give meaning and purpose) were seen as their best coping resourced against poor mental health and a pathway towards reintegration into the job market.
Ronda, Briones-Vozmediano, Galon, et al. (2015) [25]	The 2009 economic recession (Spain)	Immigrant workers from Colombia, Ecuador, Morocco, and Romania living in Madrid, Spain between February and March of 2012 (N = 44).	Six focus group discussions were held with participants in each group of the same sex and country of origin (Colombia, Ecuador, or Spain) and two key informant interviews were held with a man and woman from Romania.	During the crisis, employment opportunities were significantly reduced for men more so than women, leaving female workers to compensate for their partners’ lack of income. However, the working conditions of remaining employment also deteriorated with more temporary contracts and reduced hours, wages, and occupational health and safety protection. Participants experienced deterioration in their quality of life as a result, leading to increased stress and depression related to worries about unemployment, lower income, and debts. Frustration was felt because of limited opportunities to improve their situation and the possibility of having to relocate or return to their country of origin. Pressure and distress were associated with diffuse pain, headaches and gastric discomfort, poorer sleep, and worsening dietary habits. Family life was negatively affected by imbalanced earning between partners, leading some to marital crises, sexual problems, and relationship breakdowns. Children’s quality of life was also felt to be negatively affected. Many had worries about economic dependants in their countries of origin that provoked personal crises in immigrants’ family relationships.

**Table 2:** Summary of topics included in interview and focus group guides.

Focus Group or Interview Topics	Studies (% of All Included)
Socioeconomic changes experienced during recession or austerity	12 (92.3%)
Reactions to socioeconomic changes experienced	4 (30.8%)
Perceived changes in individual’s mental health and well-being during recession/following recession-related changes	5 (38.5%)
Consequences of changes experienced in mental health	2 (15.4%)
Narratives leading up to self-harm/suicide attempt	2 (15.4%)
Availability of/impact on informal supports, social well-being, and lifestyle of economic recession	7 (53.8%)
Help-seeking behaviors engaged in	5 (38.5%)
Challenges encountered in seeking employment	2 (15.4%)
Individual skills in managing finances	1 (7.7%)
Effects of economic crisis or austerity on the healthcare system	1 (7.7%)
Ethical dilemmas faced by healthcare providers during recession/austerity	1 (7.7%)
Access to or quality of health care/support services received	6 (46.2%)
Residual health services or other supports required	4 (30.8%)
Proposed solutions	2 (15.4%)

Note: All studies included multiple topics of inquiry; therefore, the total percentage is not equal to 100%.

## Data Availability

No new data were created or analyzed in this study. Data sharing is not applicable to this article.
